# U-blade gamma 3 vs. gamma 3 nails for intertrochanteric hip fracture: Meta-analysis

**DOI:** 10.3389/fsurg.2022.1015554

**Published:** 2022-11-23

**Authors:** Gonzalo Mariscal, Rafael Lorente, Carlos Barrios

**Affiliations:** ^1^ Institute for Research on Musculoskeletal Disorders, Valencia Catholic University, Valencia, Spain; ^2^ Department of Orthopedic Surgery, University Hospital of Badajoz, Badajoz, Spain

**Keywords:** U-Blade, gamma nail, intertrochanteric fracture, hip fracture, meta-analysis

## Abstract

**Background and Objective:**

Intertrochanteric fracture is a growing problem in the traumatology department. The use of intramedullary devices has increased, representing the first treatment option in intertrochanteric fractures. U-Blade devices appeared to avoid rotation of the femoral head over the femoral neck. The aim of this study was to conduct a meta-analysis of the surgical treatment of intertrochanteric fractures comparing in terms of safety and efficacy the U-Blade Gamma 3 nail vs. the conventional Gamma 3 nail.

**Methods:**

A literature search for intertrochanteric fracture 31A1–31A3 according to the AO foundation/orthopaedic trauma association (AO/OTA) classification was performed. Baseline characteristics of each article were obtained; radiological outcomes were tip apex distance (TAD), sliding distance (mm), cut-out rate, and lateralization rate. Surgery time (min) was also recorded. A meta-analysis was performed with ReviewManager 5.4.

**Results:**

Five retrospective studies (*n* = 993 patients) were included. With respect to TAD and sliding distance, there were no differences between two groups [mean difference (MD) 0.47, 95% confidence interval (CI), −0.46 to 1.40] and (MD 0.39, 95% CI, 0.13–0.66). The cut-out rate and lateralization rate did not show differences between two groups (*p* > 0.05). Finally, surgery time was significantly higher in the U-Blade Gamma 3 group (MD −4.84, 95% CI, −7.22 to −2.46).

**Conclusions:**

The use of U-Blade Gamma 3 did not show significant differences in the radiological results compared with the conventional Gamma 3 nail.

## Introduction

Intertrochanteric fracture is the most common fracture requiring hospitalization in elderly patients (1, 2). The management of intertrochanteric fractures remains a challenge, as many are osteoporotic patients who require multidisciplinary management with a significant economic impact (3). However, contradictory results have been reported in the surgical treatment of 31A1–31A2 intertrochanteric fractures according to the AO foundation/orthopaedic trauma association (AO/OTA) classification (4, 5).

Intramedullary nails are currently the most widely used devices in the treatment of intertrochanteric fracture (6, 7). Thus, different types of implants have been developed according to the features of each patient. U-Blade devices appeared to avoid rotation of the femoral head over the femoral neck or migration of the cephalic screw, among other complications in unstable fractures (8). U-Blade nails represent the third and most current generation of gamma nails (9). Biomechanical studies have demonstrated the efficacy of these devices compared to conventional screws. The mechanism involved is based on an increase in the surface area with the femur as well as a greater frictional strength and resistance, avoiding rotation and varus deformity, especially in osteoporotic patients (10, 11). On the other hand, only a few studies have analyzed their *in vivo* results, and there is disagreement regarding the radiological outcomes and the longer surgery time (12).

This meta-analysis aims to address a recent topic, the comparison of the results obtained with the U-Blade Gamma 3 nail and the conventional Gamma 3 nail in the treatment of intertrochanteric fractures. The use of these devices is frequent on a daily practice; however, the follow-up of the existing studies in some cases tends to be low since it is a current trend, associated with the lack of prospective studies. Therefore, the aim of this study was to conduct a meta-analysis of the surgical treatment of intertrochanteric fractures comparing in terms of safety and efficacy the U-Blade Gamma 3 nail vs. the conventional Gamma 3 nail.

## Materials and methods

### Information sources and eligibility criteria

**Figure 1:**
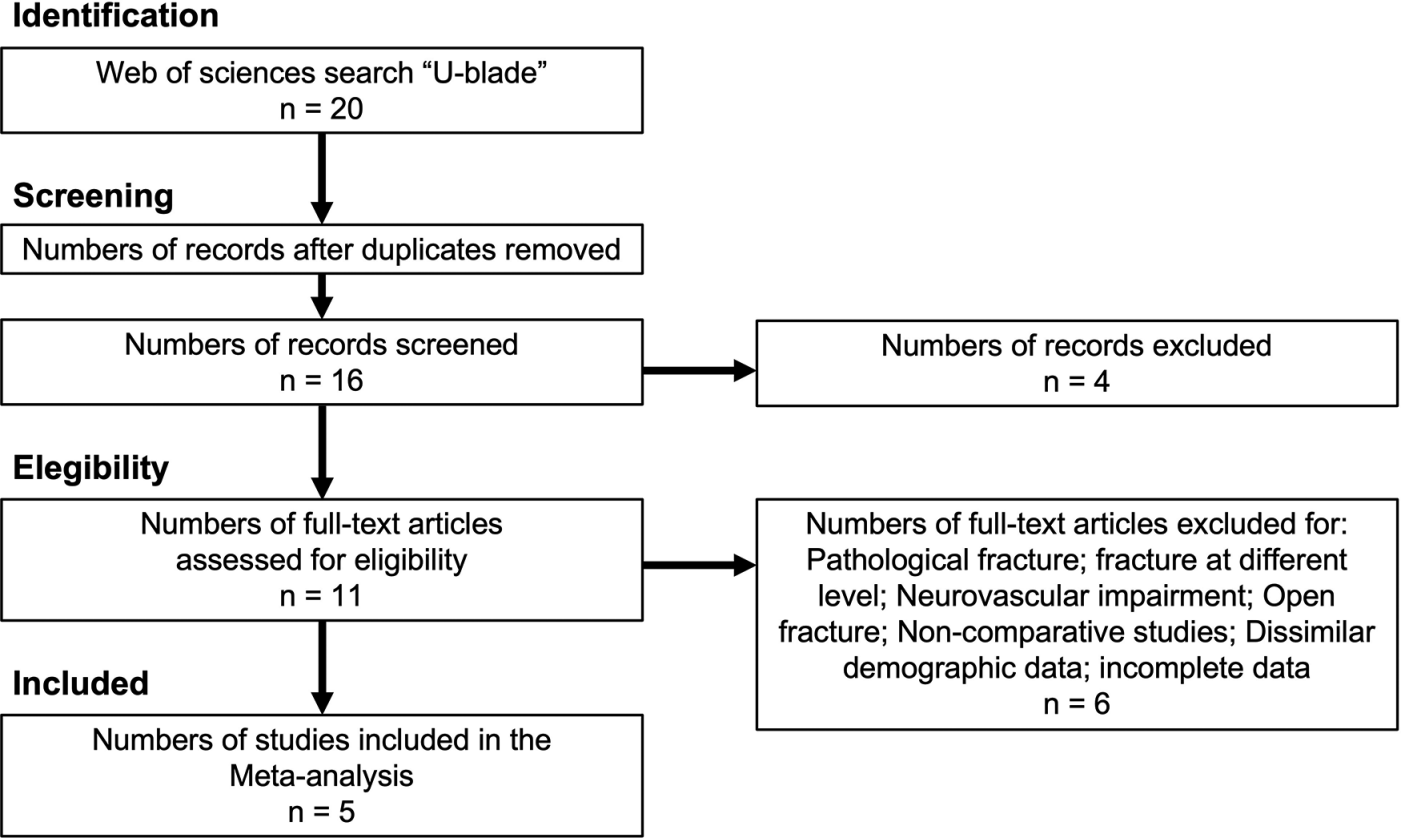
Study selection flow diagram (Preferred Reporting Items for Systematic reviews and Meta-Analysis).

The current study followed PRISMA guidelines (13) (Figure 1). Language was limited to English. The research question was conducted following the PICOS strategy: (P) patients with intertrochanteric fracture classified according to AO/OTA 31A1–31A3; (I) interventions were U-Blade Gamma 3 intramedullary short nail vs. conventional Gamma 3 short intramedullary nail; (C) comparisons were the efficacy and safety of the interventions; (O) outcomes were tip apex distance (TAD) postoperative, sliding distance at 1 year, n of patients presented lateralization, cut-out, and time of surgery; (S) we included cohort retrospective studies. The diagnosis of the fractures was made by x-rays or CT. We excluded patients younger than 65 years, high-energy trauma, pathologic fracture, follow-up less than 6 months, additional surgeries, coexistence of fracture at different location, bilateral intertrochanteric fracture, neurovascular alteration, open fracture, duplicated data, incomplete data, or case series studies. In studies that included three arms of comparisons, the arms of interest were selected.

### Search methods for identification of studies

The search strategy was the following: (“U-Blade”). Two authors independently reviewed the studies. An initial screening of titles and abstracts was performed to eliminate studies that were obviously outside the scope of the review. In cases of uncertainty based on title or abstract, the full text of each article was examined for further evaluation. If consensus was not reached, a third review author was asked to complete the data extraction form and discuss the article with the other two authors until consensus was reached. All disagreements were resolved by discussion. A systematic search of the literature using PubMed, EMBASE, Scopus, and the Cochrane Collaboration Library database was carried out. No date limit was specified as this was a recent topic.

### Data extraction and data items

The baseline characteristics of each study were obtained: number of participants, type of study, type of fracture according to the AO/OTA classification, length of nail, diagnosis method, follow-up (minimum follow-up), age, and gender. Only outcomes that were given for at least three studies were considered. The main radiological outcomes were TAD postoperative (mm), sliding distance at 1 year (mm), and n of patients presented lateralization and cut-out. We also recorded the time of surgery (min).

### Assessment of risk of bias in included studies

The methodological quality of the studies was independently assessed by two reviewers using the Newcastle–Ottawa Quality Assessment Scale for Cohort Studies (14). This scale examines participant selection and study design, comparability of groups, and exposure/outcome ascertainment. Based on their score, studies were classified as low quality (0–3 points), moderate quality (4–6), or high quality (7–9) (Table 1).

**Table 1:** Newcastle–Ottawa quality assessment scale—cohort studies.

Study	Representativeness of the exposed cohort	Selection of the nonexposed cohort	Ascertainment of exposure	Demonstration that outcome of interest was not present at start of study	Comparability of cohorts on the basis of the design or analysis controlled for confounders	Assessment of the outcome	Was follow-up long enough for outcomes to occur	Adequacy of follow-up of cohorts	Total
Ryu et al. (15)	*	*	*	*	*	*	*	*	8
Kang et al. (16)	—	*	*	*	*	*	*	*	7
Oh et al. (17)	*	*	*	*	*	*	*	*	8
Lang et al (18)	*	*	*	*	*	*	*	*	8
Lang et al. (19)	*	*	*	*	*	*	—	*	7

### Assessment of results

The meta-analysis was performed using the ReviewManager 5.4 software package provided by the Cochrane Collaboration. For dichotomous variables, odds ratios with a confidence interval (CI) of 95% were calculated. The weighted mean difference (MD) and the 95% CI were calculated for the continuous variables. Heterogeneity was checked with both the *χ*^2^ and the *I*^2^ test. *I*^2^ varies from 0% to 100%, considering the values of 25%, 50%, and 75% as low, moderate, and high heterogeneity, respectively. A fixed-effects model was adopted if there was no statistical evidence of heterogeneity, and a random-effects model was adopted if significant heterogeneity was observed. Publication bias was evaluated using the funnel plot diagrams.

## Results

### Types of interventions

Groups were U-Blade Gamma 3 intramedullary short nail vs. conventional Gamma 3 short intramedullary nail. Different types of nails length were included, varied from 170 to 200 mm (Table 1). Distal fixation was used. Surgery was performed by a specialist. The surgical position was supine, guided by fluoroscopy.

### Description of studies

Table 2 shows the characteristics of the included studies (15–19). Five retrospective cohort studies were included. There was a pool of 993 patients. Mean follow-up time ranged from 1 to 6 years. Age ranged from 80 to 84 years old, and the overall percentage of females ranged from 67% to 87%.

**Table 2:** Characteristics of the included studies.

Study	*N*		Type of study	Fracture AO/OTA	Stable/nonstable		Length (mm)	Countries	Manufacturers	Diagnosis	Follow-up (years)	Mean age (SD)	% Female
	Gamma 3	U-Blade Gamma 3			Gamma 3	U-Blade Gamma 3							
Ryu et al. (15)	72	90	Retrospective	31A1–31A3	37/35	47/43	—	Korea	Stryker	X-rays	3	82.4 (6.4)	82.1 (6.4)	71.0%
Kang et al. (16)	53	53	Retrospective	31A2, 31A3	0/53	0/53	—	Republic of Korea	Zimmer, Synthes, Stryker	CT	6	81.3 (9.4)	79.7 (10.7)	69.8%
Oh et al. (17)	152	152	Retrospective	31A1- 31A3	66/86	66/86	170	Republic of Korea	Stryker	X-rays	4	80.7 (7.6)	81.4 (7.0)	67.4%
Lang et al (18)	135	135	Retrospective	31A1- 31A3	54/81	54/81	200	Austria	Stryker	X-rays	5	83.1	83.5	87.4%
Lang et al. (19)	82	69	Retrospective	31A2	—	—	200	Austria	Stryker	X-rays	1	82.0 (10.0)	83.2 (9.6)	80.1%

### Effects of interventions

**Figure 2:**
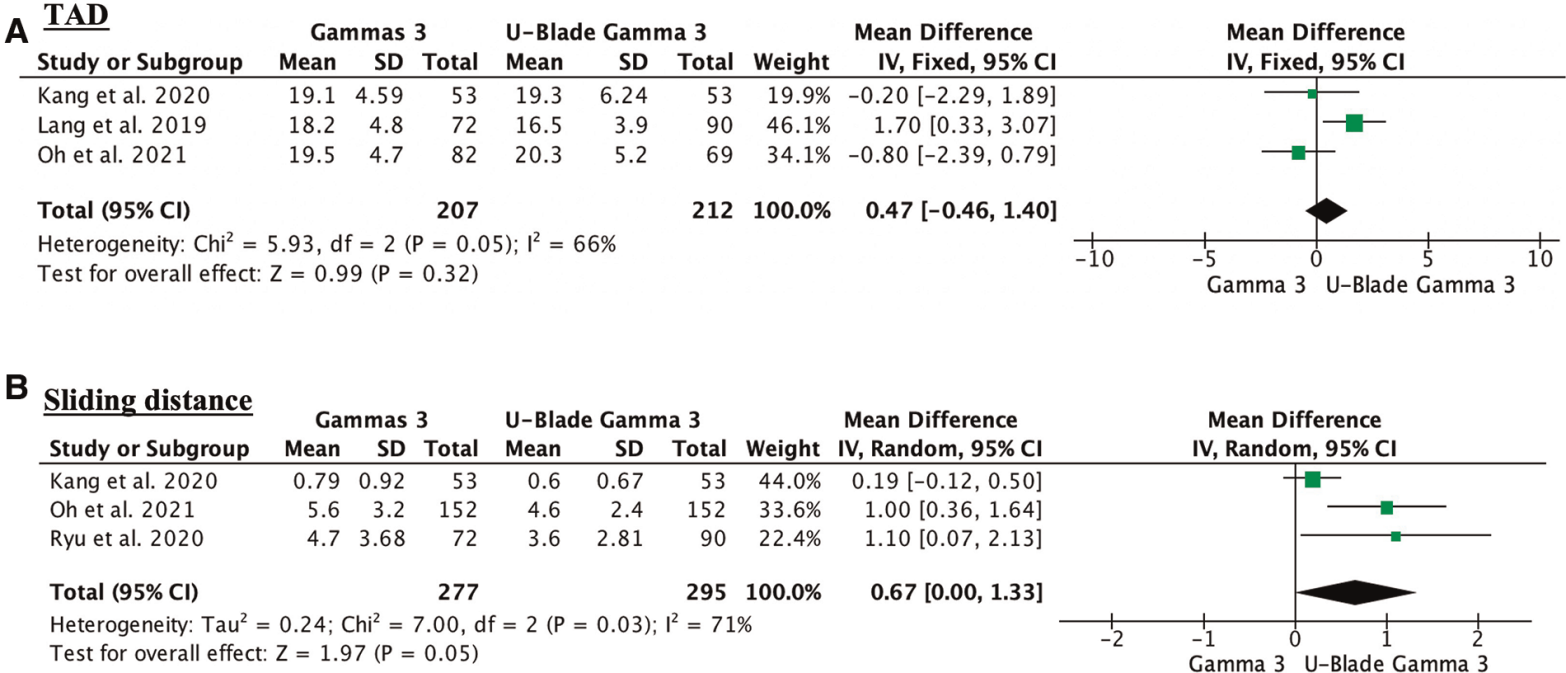
(**A**) Forest plot showing the TAD outcome. The conventional Gamma 3 presented a higher TAD in one of the three studies analyzing this outcome but there were no significant differences between groups. (**B**) Forest plot showing no differences regarding the sliding distance between groups. There was also a high heterogeneity (*I*^2^ = 71%).

**Figure 3:**
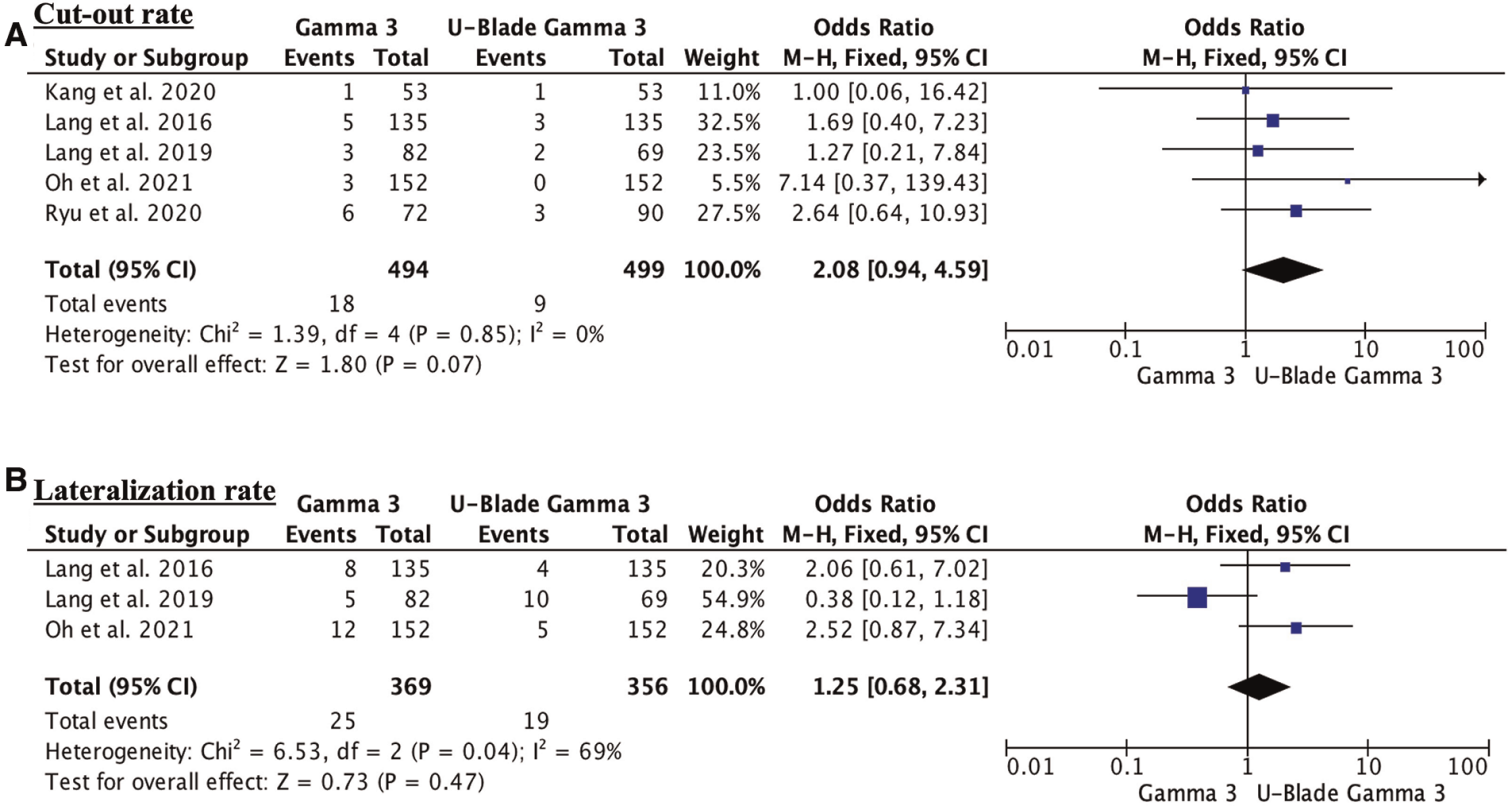
(**A**) Forest plot showing the cut-out rate. There were no differences between groups. Despite all studies except one supporting the U-Blade, care must be taken when evaluating the findings of individual studies given the potential for selection bias. (**B**) Forest plot showing the lateralization rate. The lateralization rate outcome showed substantial heterogeneity (*I*^2^ = 69%).

**Figure 4:**
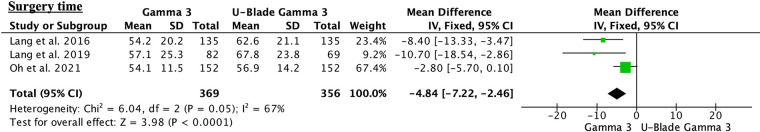
Forest plot showing the surgery time. Significant differences were observed in favor of conventional Gamma 3 (*p* < 0.0001), but the mean difference was 4.84 min less than U-Blade Gamma 3.

Respect to TAD and sliding distance there were no differences between two groups [(MD 0.47, 95% CI, −0.46 to 1.40; participants = 419; studies = 3; *I*^2^ = 66%; Figure 2A) and (MD 0.67, 95% CI, 0.00–1.33; participants = 572; studies = 3; *I*^2^ = 71%; Figure 2B)]. The lateralization rate did not show significant differences between two groups (odds ratio (OR) 1.25, 95% CI, 0.68–2.31; participants = 725; studies = 3; *I*^2^ = 69%) (Figure 3A). The cut-out was higher in the Gamma 3 conventional group but there were no significant differences (OR 2.08, 95% CI, 0.94–4.59; participants = 993; studies = 5; *I*^2^ = 0%) (Figure 3B). Finally, the surgery time was significantly higher in the U-Blade Gamma 3 group (MD −4.84, 95% CI, −7.22 to −2.46; participants = 725; studies = 3; *I*^2^ = 67%) (Figure 4).

### Sensitivity analysis

**Figure 5:**
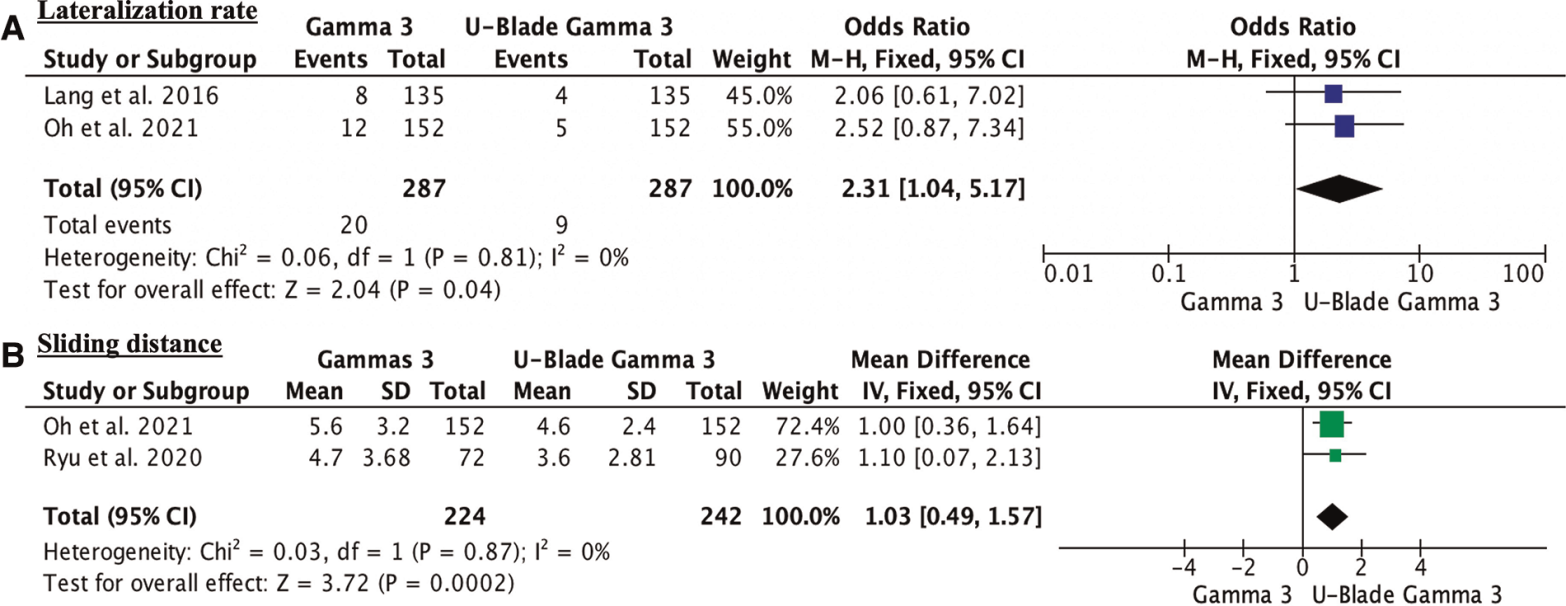
(**A**) sensitivity analysis showing statistically significant differences regarding the lateralization rate. (**B**) Sensitivity analysis showing statistically significant differences regarding the sliding distance.

After eliminating the top-weight study from the comparisons in all the outcomes, two of the variables became statistically significant. These outcomes were the lateralization and sliding distance (Figure 5).

## Discussion

This meta-analysis compares the U-Blade Gamma 3 nail with the conventional Gamma 3 nail in the treatment of AO/OTA 31A1–31A3 intertrochanteric fractures. All studies were retrospective so the results should be considered with caution. This meta-analysis found no differences in radiological outcomes assessed by TAD, sliding distance, cut-out, and lateralization. However, significant differences were observed in surgery time.

The quality of the evidence was low since most of the included studies were retrospective studies with level III evidence. It was also not possible to perform a blinding process for obvious reasons in both surgeons and patients. Since this was a novel topic, there were not enough published studies, so the number of comparisons in some variables was limited. For this reason, only outcomes from at least three studies were included. Follow-up according to the outcomes differed among the studies, which compromised the greater number of comparisons and variables included in this meta-analysis.

Potential biases in the review process were low as all studies included a similar structure and used the same variable definitions. Risk of bias in the diagnosis of fractures could exist since in one study, CT was used in comparison with conventional x-rays, resulting in potential inclusion biases. Nevertheless, all types of stable and unstable fractures were included. To the best of our knowledge, this is the first meta-analysis on this topic, so comparisons with similar studies could not be established.

There were no significant differences in postoperative TAD and sliding distance at one year. Lang et al. included patients with unstable type 31A2 fractures and was the only study that reported significant differences regarding these outcomes (19). Lang et al. did not observe radiological differences (18). It would be of interest to compare TAD with a higher follow-up since it is considered one of the most important predictors of failure, but it was not possible since this variable was provided by less than three studies (20). In addition, the sliding distance has been related to the shortening of the limb vs. the contralateral limb, so the inclusion of results from both limbs would be relevant.

Regarding the cut-out and lateralization rate, it seems expected to be lower with the U-Blade nail, but there were no significant differences in the lateralization and cut-out rate. The cut-out rate with the conventional Gamma 3 nail and the U-Blade Gamma 3 was 3.6% and 1.8% respectively, which is in accordance with the literature (8, 21). Lang et al. showed a lower rate of cut-out with U-Blade Gamma 3 nails (18). This point could be critical for fracture healing by providing greater strength and stability.

Finally, surgery time was longer in the U-Blade Gamma 3 group. This finding could be expected as the nail includes additional steps in surgery and the use of the U-nail is unfamiliar, which leads to a long surgery time. This may affect patient safety and operating room efficiency (22).

Some of the limitations are as follows: All the studies were retrospective. Indeed, a selection bias may occur since it was not possible to establish the criteria for the use of U-Blade by the surgeon at the time of surgery. It was also not possible to identify subgroups, since there were no studies that analyzed only stable fractures. Most of the fractures were nonstable and the proportion of the nonstable fracture in each treatment group was similar. No further exploration of statistical heterogeneity with subgroup analysis has been attempted due to the limited number of articles, thus limiting our confidence in the validity of the study results. In addition, there were a lack of variables with a greater follow-up, complications, and functional results. However, there was homogeneity regarding the use of different nail sizes and the same variable definitions. Most studies did not provide detailed information on the identity and age of the operator. One of the studies showed that all surgeries were performed by the same surgeon with more than 20 years of experience. The second study showed that the surgeries were performed by three orthopedic surgeons specialized in hip surgery.

In conclusion, the use of U-Blade Gamma 3 did not show significant differences in the radiological results with respect to the conventional Gamma 3 nail. This fact, along with the longer surgery time and higher cost of the U-Blade compared with the conventional nail, does not justify their use. This meta-analysis could help orthopedic surgeons to choose the appropriate type of device.

## Data Availability

The original contributions presented in the study are included in the article/Supplementary Material, further inquiries can be directed to the corresponding author.
